# RhoGDIβ promotes Sp1/MMP‐2 expression and bladder cancer invasion through perturbing miR‐200c‐targeted JNK2 protein translation

**DOI:** 10.1002/1878-0261.12132

**Published:** 2017-09-11

**Authors:** Haishan Huang, Honglei Jin, Huirong Zhao, Jingjing Wang, Xin Li, Huiying Yan, Shuai Wang, Xirui Guo, Lei Xue, Jingxia Li, Minggang Peng, Annette Wang, Junlan Zhu, Xue‐Ru Wu, Changyan Chen, Chuanshu Huang

**Affiliations:** ^1^ Zhejiang Provincial Key Laboratory for Technology & Application of Model Organisms School of Laboratory Medicine and Life Science Wenzhou Medical University China; ^2^ Nelson Institute of Environmental Medicine New York University School of Medicine Tuxedo NY USA; ^3^ Departments of Urology New York University School of Medicine NY USA; ^4^ The Center of Drug Discovery Northeastern University Boston MA USA

**Keywords:** bladder cancer, invasion, miR‐200c, RhoGDIβ

## Abstract

Our most recent studies demonstrate that RhoGDIβ is able to promote human bladder cancer (BC) invasion and metastasis in an X‐link inhibitor of apoptosis protein‐dependent fashion accompanied by increased levels of matrix metalloproteinase (MMP)‐2 protein expression. We also found that RhoGDIβ and MMP‐2 protein expressions are consistently upregulated in both invasive BC tissues and cell lines. In the present study, we show that knockdown of RhoGDIβ inhibited MMP‐2 protein expression accompanied by a reduction of invasion in human BC cells, whereas ectopic expression of RhoGDIβ upregulated MMP‐2 protein expression and promoted invasion as well. The mechanistic studies indicated that MMP‐2 was upregulated by RhoGDIβ at the transcriptional level by increased specific binding of the transcription factor Sp1 to the *mmp‐2* promoter region. Further investigation revealed that RhoGDIβ overexpression led to downregulation of miR‐200c, whereas miR‐200c was able directly to target 3′‐UTR of *jnk2* mRNA and attenuated JNK2 protein translation, which resulted in attenuation of *Sp1* mRNA and protein expression in turn, inhibiting Sp1‐dependent *mmp‐2* transcription. Collectively, our studies demonstrate that RhoGDIβ overexpression inhibits miR‐200c abundance, which consequently results in increases of JNK2 protein translation, Sp1 expression, *mmp‐2* transcription, and BC invasion. These findings, together with our previous results showing X‐link inhibitor of apoptosis protein mediating mRNA stabilization of both *RhoGDIβ* and *mmp‐2*, reveal the nature of the MMP‐2 regulatory network, which leads to MMP‐2 overexpression and BC invasion.

**Abbreviations**
BBN: *N*‐butyl‐*N*‐(4‐hydroxybutyl) nitrosamine
BC: bladder cancer
CHX: cycloheximide
EMT: epithelial‐to‐mesenchymal transition
GDP: guanosine diphosphate
GTP: guanosine triphosphate
IHC: immunohistochemistry staining
ISO: isorhapontigenin
MIBC: muscle‐invasive bladder cancer
MMP: matrix metalloproteinase
NMIBC: non‐muscle‐invasive bladder cancer
RhoGDI: RhoGDP dissociation inhibitor
XIAP: X‐link inhibitor of apoptosis protein

## 1 Introduction

In China, approximately 80 500 cases of bladder cancer (BC) are diagnosed with roughly 32 900 cancer‐related deaths in year 2015 (Chen *et al*., 2016). BC is also one of the most common human malignancies worldwide (Siegel *et al*., 2016). The majority of BC could be classed into two major clinicopathological phenotypes: muscle‐invasive bladder cancer (MIBC) and non‐muscle‐invasive bladder cancer (NMIBC) (Blaveri *et al*., 2005; Choi *et al*., 2014a; Damrauer *et al*., 2014; Sjödahl *et al*., 2012). At least 50% of the patients with muscle‐invasive BC die from metastases within 2 years, and the 5‐year survival rate for metastatic bladder cancer is only 6% (Choi *et al*., 2014a). MIBCs are biologically heterogeneous and have widely variable clinical outcomes and responses to conventional chemotherapy (Choi *et al*., 2014b). Development of refractory bladder tumors is closely correlated with the degree of the ability for cancer invasion or metastasis (Kaufman *et al*., 2009). In light of this information, it becomes evident that there is an imminent need for identification of the intracellular molecules responsible for bladder cancer metastasis and the subsequent target of these molecules for bladder cancer therapeutics (Funt and Rosenberg, 2017; Gorin *et al*., 2016) .

The RhoGDI family has been reported to be involved in the control of cancer invasion and metastasis; it consists of three members, RhoGDIα, RhoGDIβ and Rho‐GDIγ, which are well known for their regulation of the guanosine diphosphate (GDP)/guanosine triphosphate (GTP) exchange (Duckett *et al*., 1998). RhoGDIα is expressed ubiquitously in many cells and tissues (Sasaki and Takai, 1998), whereas RhoGDIβ commonly exists in hematopoietic, endothelial, and urothelial cells (Sasaki and Takai, 1998). Our published studies have revealed that X‐link inhibitor of apoptosis protein (XIAP), through its RING domain, is able to associate with RhoGDIα and inhibit RhoGDIα SUMOylation, by which XIAP inhibits colorectal cancer cell mobility (Fang *et al*., 2012). Our most recent studies also demonstrate that RhoGDIβ functions as a downstream mediator of XIAP and mediates matrix metalloproteinase (MMP)‐2 expression, which positively regulates bladder cancer invasion (H. Jin and C. Huang, unpublished data). However, the mechanisms underlying overexpressed RhoGDIβ leading to *mmp‐2* transcription and cancer invasion of human BC have never been explored.

Hydration of the extracellular matrix (ECM) is an important process that allows cancer cell invasion and metastasis through the secretion of enzymes such as MMP‐2 and MMP‐9 (Jacob and Prekeris, 2015). MMP‐2 (72 kD type IV collagenase) possesses enzymatic activity that mediates degradation of type IV collagen, which is a major structural component of the basement membrane of tissues (Mook *et al*., 2004). Alterations of MMP‐2 are often associated with the metastasis of many other cancers, including colorectal cancer (Miao *et al*., 2015), ovarian cancer (Zhuang *et al*., 2015), and breast cancer (Miao *et al*., 2015). Our recent study reveals that MMP‐2 overexpression is crucial for human BC invasion (Jin *et al*., 2015), whereas inhibition of MMP‐2 expression by the anti‐cancer agent isorhapontigenin (ISO) dramatically attenuated both BC invasion *in vitro* and highly invasive BC formation *in vivo* (Jiang *et al*., 2016) .

MicroRNA (miRNA) is a kind of non‐coding RNA that has a regulatory function on coding miRNA by binding to the 3′‐UTR of coding mRNA, by which miRNA modulates mRNA stability or protein translation (Enokida *et al*., 2016; Liu and Gao, 2016). miRNA is therefore considered to be either oncogenic or antineoplastic, depending on its downstream target mRNA (Enokida *et al*., 2016). MiR‐200c is considered to be an important regulator that is involved in suppression of tumorigenesis or cancer metastasis (Berlanga *et al*., 2016; Jiao *et al*., 2016; Sui *et al*., 2014). In the present study, by using loss‐ and gain‐strategy, we discovered that RhoGDIβ is able to attenuate miR‐200c abundance, which releases its inhibition of JNK2 protein translation, resulting in specific JNK2 induction, in turn leading to Sp1 protein expression, and promoting *mmp‐2* transcription, consequently increasing BC cell invasion.

## 2 Materials and methods

### 2.1 Cell lines, plasmids, and antibodies

Human BC T24 and T24T cells have been used in the current studies. T24 is a non‐metastatic cell line, whereas T24T is a BC cell line with lung metastatic ability (Gildea *et al*., 2000; Grossman *et al*., 1986). Both cell lines were cultured in DMEM:F‐12 1 : 1 with 10% fetal bovine serum (FBS; ATLANTA, Flowery Branch, GA, USA) as described in our previous studies (Jiang *et al*., 2016; Jin *et al*., 2015), and cell lines will be authenticated every 6–12 months by Genetica DNA Laboratories (Burlington, NC 27215, CA, USA) and the results compared with the data in the ATCC STR database. The human *mmp‐2* promoter‐driven luciferase reporter was a gift from Dr. Yi Sun (Michigan University, MI, USA) (Qin *et al*., 1999). The mutation of *mmp‐2* promoter‐driven luciferase at the Sp1 binding site was made by point mutation using the following primers: Sense: 5′‐GTA GGG GGG TGG GGC AGA GAG ATA CGG GCC CGA GTG CGC CC‐3′ anti‐sense: 5′‐GGG CGC ACT CGG GCC CGT ATC TCT CTG CCC CAC CCC CCT AC‐3′. The Sp1‐dependent luciferase reporter, which contains the three consensus binding sites of Sp1, has been described previously (Fang *et al*., 2012). The short hairpin RNA constructs specific to Sp1(shSp1), JNK2(shJNK2), MMP‐2(shMMP‐2), and their scramble nonsense control construct were purchased from Open Biosystem (Pittsburgh, PA, USA). The antibodies for MMP‐2, RhoGDIβ, E2F1, HSF1, ETS‐1, p53, Smad4, Sp1, and β‐actin, were purchased from Santa Cruz Biotechnology, Inc. (Santa Cruz, CA, USA). The antibodies against phosphor‐c‐Jun (p‐c‐Jun) at Ser63, p‐c‐Jun at Ser73, C‐Jun, JNK1, JNK2, phosphor‐JNK (p‐JNK) at Thr183/Tyr185, extracellular signal‐regulated kinases (ERK), phosphor‐ERK (p‐ERK) at Thr202/Tyr204, p38, phosphor‐p38 (p‐p38) at Thr180/Tyr182, and GAPDH were bought from Cell Signaling Technology (Beverly, MA, USA).

### 2.2 Cell transfection

Cell transfections were performed with PolyJet™ DNA in Vitro Transfection Reagent (SignaGen Laboratories, Rockville, MD, USA) according to the manufacturer's instructions. For stable transfection, cell cultures were subjected to selection with hygromycin B (200–400 μg·mL^−1^), G418 (500–1000 μg·mL^−1^) or puromycin (0.2–0.3 μg·mL^−1^), depending on the different antibiotic resistance plasmids transfected. Cells surviving the antibiotics selection were pooled as stable mass transfectants.

### 2.3 Western blot analysis

Whole cell extracts were prepared using cell lysis buffer (10 mm Tris‐HCl, pH 7.4, 1% SDS, and 1 mm Na_3_VO_4_) as described in our previous studies (Yu *et al*., 2014; Zhang *et al*., 2014). Proteins were resolved by SDS/PAGE, transferred to a membrane, probed with primary antibodies, and incubated with the alkaline phosphatase (AP)‐conjugated secondary antibody. Signals were detected by the enhanced chemifluorescence western blotting system as described in our previous report (Xie *et al*., 2016). The images were acquired by scanning with the phosphoimager Typhoon FLA 7000 (GE, Pittsburgh, PA, USA).

### 2.4 *N*‐Butyl‐*N*‐(4‐hydroxybutyl) nitrosamine (BBN)‐induced mouse bladder cancer model and immunohistochemistry paraffin (IHC‐P)

All animal procedures were approved by the Committee on Animal Resources of the New York University School of Medicine in accordance with NIH guidelines. The C57BL/6 male mice (*n *=* *12/group) aged 3–4 weeks were supplied *ad libitum* with tap water containing 0.05% BBN (TCI America, Portland, OR, USA) in opaque bottles for 23 weeks, while negative control mice received regular tap water. The drinking water was prepared freshly twice a week, and consumption was recorded to estimate BBN intake. Mice were sacrificed at week 23 of the experiments and bladders were harvested and preserved in paraffin for pathological analysis and immunohistochemistry staining (IHC). Bladder tissues obtained from the sacrificed mice specimens were formalin‐fixed and paraffin‐embedded. IHC was performed to evaluate JNK2 expression in both BBN‐induced invasive bladder cancer tissues and negative control bladder tissues using antibodies specific against JNK2 (Cell Signaling Technology) together with an IHC kit based on the protocol instruction, as described in our previous studies (Xie *et al*., 2016; Zhang *et al*., 2015). The resultant immunostaining images were captured using the axiovision rel.4.6 computerized image analysis system (Carl Zeiss, Oberkochen, Germany). JNK2 protein expression levels were analyzed by calculating the integrated optical density per stained area (IOD/area) using imagepro plus version 6.0 ( Media Cybernetics, Bethesda, MD, USA).

### 2.5 Luciferase reporter assay

Each luciferase reporter construct tested, together with pRL‐TK, was transiently transfected into the indicated cells. Twenty‐four hours after the transfection, luciferase activity was determined using the luciferase Assay System kit (Promega, Madison, WI, USA) as described in our previous studies (Liang *et al*., 2016; Xie *et al*., 2016). The results were normalized by internal TK signal. All experiments were done in triplicate and the results are expressed as mean ± SD (standard error).

### 2.6 RT‐PCR and real‐time PCR for mRNA

Total RNA was extracted using the TRIzol reagent (Invitrogen, Grand Island, NY, USA) as per the manufacturer's instructions. Total RNA 5 μg was used for first‐strand cDNA synthesis with oligdT primer by SuperScript™ First‐Strand Synthesis system (Invitrogen). PCR amplification was carried out using specific primers (Invitrogen). The primers used in this study were: *human mmp‐2* (Forward: 5′‐caa gtg gga caa gaa cca ga‐3′, Reverse: 5′‐cca aag ttg atc atg atg tc‐3′), *human sp1* (Forward: 5′‐att aac ctc agt gca ttg ggt a‐3′, Reverse: 5′‐agg gca ggc aaa ttt ctt ctc‐3′), *human jnk2* (Forward: 5′‐atg aag aaa ctt cag cca act gt‐3′, Reverse: 5′‐aca gat ctc tgg ctt gac tt ‐3′) and *human gapdh* (Forward: 5′‐gat gat ctt gag gct gtt gtc‐3′, Reverse: 5′‐cag ggc tgc ttt taa ctc tg‐3′). Real‐time PCR was conducted following the protocol for Fast SYBR Green Master Mix kit (Applied Biosystems, Foster City, CA, USA; 4385614) in the 7900HT Fast Real‐Time PCR System (Applied Biosystems) using the same cDNs used for RT‐PCR as described in our previous publication (Huang *et al*., 2016).

### 2.7 Quantitative RT‐PCR for miRNA assay

Total microRNA was extracted using the miRNeasy Mini Kit (Qiagen, Valencia, CA, USA). Total RNA (1 μg) was used for reverse transcription. Analysis of miR‐203, miR‐106a, miR‐106b, miR‐429, miR‐93, miR‐20a, miR‐20b, miR‐17, miR‐200c, miR‐125, miR‐320a, miR‐320b, miR‐320c, miR‐320d, and miR‐4429 expression was conducted using the 7900HT Fast Real‐time PCR system (Applied Biosystems) with the miScript PCR kit (Qiagen). The primers for microRNA were purchased from Invitrogen, and U6 was used as a control. Cycle threshold (CT) values were determined, and the relative expression of microRNA was calculated using the values of 2^−ΔΔCT^.

### 2.8 Cell invasion assay

The invasion assay was performed using BD BioCoat™ Tumor Invasion System (BD Falcon, NY, USA) according to the manufacturer's instructions. Briefly, cells (3 × 10^4^) were simultaneously seeded onto inserts of the chambers coated (invasion) or uncoated (migration) with Matrigel™ matrix in triplicate, in 400 μL of serum‐free F12/DMEM medium. Inserts were placed into wells containing 1 mL of medium supplemented with 10% FBS. The cells were incubated for 24 h and the cells on the upper surface of the filters were completely removed by wiping with a cotton swab. The inserts were then fixed with 3.7% formalin and subsequently with 100% methanol, and stained with Giemsa. The photographs were taken with an Olympus DP71, and the numbers of migrated cells attached to the other side of the insert were counted under a light Olympus DP71 microscope in eight random fields at a magnification of ×200. The number of migrated and invasive cells per image was determined using imagej software (National Institutes of Health, Bethesda, MD, USA). Data is presented as the percentage of invasion through the BD Matrigel™ matrix and membrane relative to the migration of cells through uncoated membrane. The data shown are representative of three independent experiments.

### 2.9 Statistical analysis

Student's *t*‐test was utilized to determine significant differences. The differences were considered to be significant at *P* ≤ 0.05.

## 3 Results

### 3.1 MMP‐2 functions as the RhoGDIβ downstream effector for BC invasion

**Figure 1:**
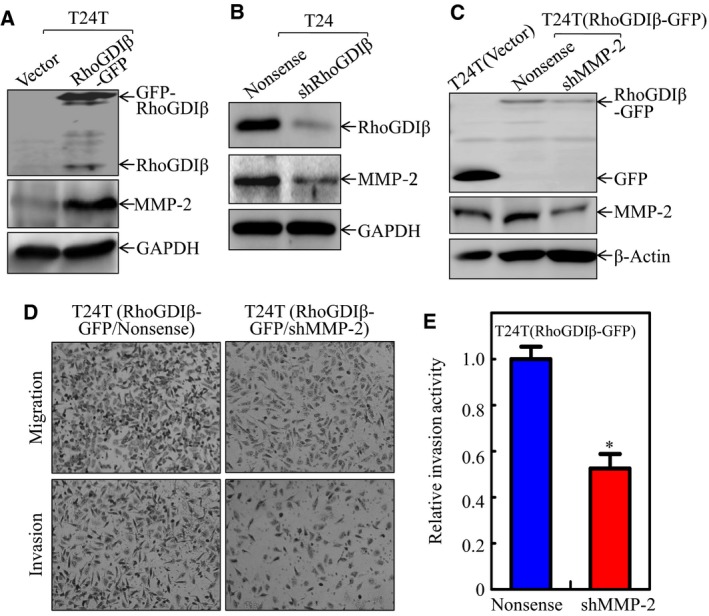
MMP‐2‐mediated RhoGDIβ promoting BC invasion. (A–C) RhoGDIβ and MMP‐2 expressions were analyzed by western blot (WB) in (A) T24T(RhoGDIβ‐GFP) *vs*. T24T(Vector) cells, (B) T24(shRhoGDIβ) *vs*. T24(Nonsense) and (C) T24T(Vector) *vs*. T24T(RhoGDIβ‐GFP/Nonsense) and T24T(RhoGDIβ‐GFP/shMPP‐2). (D, E) Invasive abilities of T24T(RhoGDIβ‐GFP/shMMP‐2) cells and T24T(RhoGDIβ‐GFP/Nonsense) were determined using BD BioCoat™Matrigel™ Invasion Chamber (D), and the relative invasion activity in these two transfectants was plotted (E). *Significant inhibition of invasion in comparison with T24T(RhoGDIβ‐GFP/Nonsense) cells (*P* < 0.05).

T24 is a high‐grade invasive BC with non‐metastatic ability, whereas T24T is derived from T24 cells by mouse metastatic screening and therefore possesses a high metastastic ability for lung (Gildea *et al*., 2000). Thus, T24 and T24T cells are unique, well‐established cellular experimental models for exploring the function of the specific gene in BC invasion and metastasis. To identify the RhoGDIβ downstream effectors during the process of promoting BC cell invasion, we stably introduced RhoGDIβ‐GFP into T24T cells. The stable transfectant, T24T (RhoGDIβ‐GFP), and its vector control transfectant, T24T(Vector), were established and the ectopic expression of RhoGDIβ was validated by western blot, as shown in Fig. 1A. Since our published studies indicate that MMP‐2 is critical for human BC invasion (Jin *et al*., 2015), we then evaluated the effect of RhoGDIβ overexpression on MMP‐2 expression. The overexpression of RhoGDIβ‐GFP resulted in a remarkable upregulation of MMP‐2 protein in comparison with that in T24T(Vector) cells. Consistently, stable knockdown of RhoGDIβ by its specific shRNA caused a dramatic attenuation of MMP‐2 expression in T24 cells (Fig. 1B). Next, we knocked down *mmp‐2* in T24T(RhoGDIβ‐GFP) cells to evaluate the effect of MMP‐2 on T24T cell invasion. As shown in Fig. 1C–E, the knockdown of *mmp‐2* impaired BC invasion, suggesting that MMP‐2 is a RhoGDIβ downstream effector that might be responsible for promoting BC cell invasion.

### 3.2 RhoGDIβ upregulated *mmp‐2* mRNA transcription

**Figure 2:**
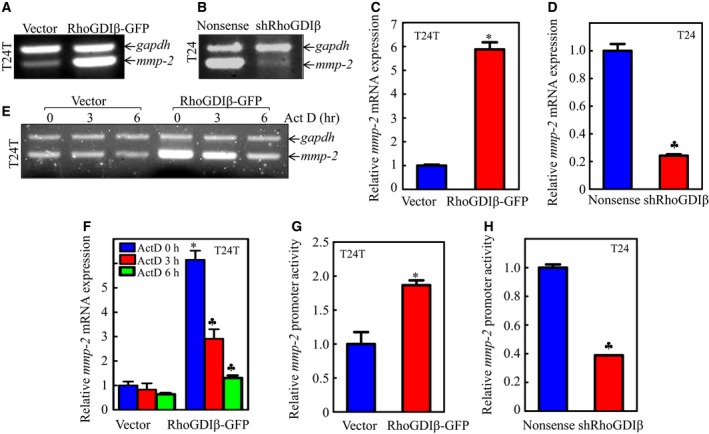
The gene *mmp‐2* was upregulated at the transcriptional level by RhoGDIβ. (A–F) Total RNA was extracted from the cells, as indicated. After reverse transcription, the mRNA levels of *mmp‐2* in cells were evaluated using RT‐PCR assay (A, B) and real‐time PCR assay (C, D). *mmp‐2* mRNA stabilities were evaluated by regular RT‐PCR (E) or real‐time PCR (F) in the presence of Act D in both T24T(Vector) and T24T(RhoGDIβ‐GFP) cells. (G, H) Wild‐type *mmp‐2* promoter‐driven luciferase reporter was co‐transfected together with pRL‐TK into T24T(Vector) and T24T(RhoGDIβ‐GFP) cells (G), or T24(Nonsense) and T24(shRhoGDIβ) cells (H), respectively. Twenty‐four hours post‐transfection, the luciferase activity was evaluated. TK was used as internal control. The results are presented as mmp‐2 promoter activity relative to the control vector transfectant, and each bar indicates mean ± SD from three independent experiments. *Significant increase (*P* < 0.05). ♣Significant inhibition.

To investigate whether RhoGDIβ promoted MMP‐2 expression at the mRNA level, we tested the mRNA abundance of *mmp‐2* in T24T(RhoGDIβ‐GFP) *vs*. T24T(Vector) and T24(shRhoGDIβ) *vs*. T24(Nonsense) cells. The patterns of mRNA expressions were consistent with the changes of MMP‐2 protein expression (Fig. 2A–D). The results from the investigation of *mmp‐2* mRNA stability using reverse transcription PCR (RT‐PCR) or real‐time PCR showed that the half‐life of *mmp‐2* mRNA in T24T(RhoGDIβ‐GFP) cells was much shorter than that in T24T(Vector) cells (Fig. 2E, F), suggesting that RhoGDIβ overexpression results in unstable *mmp‐2* mRNA. We then evaluated the *mmp‐2* promoter transcriptional activity using an *mmp‐2* promoter‐driven luciferase reporter. The results indicated that overexpression of RhoGDIβ augmented *mmp‐2* promoter activity (Fig. 2G), whereas RhoGDIβ knockdown showed the opposite effect (Fig. 2H). Taken together, our results suggest that RhoGDIβ exerts its upregulation of MMP‐2 at the mRNA transcription level.

### 3.3 Sp1 was responsible for RhoGDIβ upregulation of *mmp‐2* transcription

**Figure 3:**
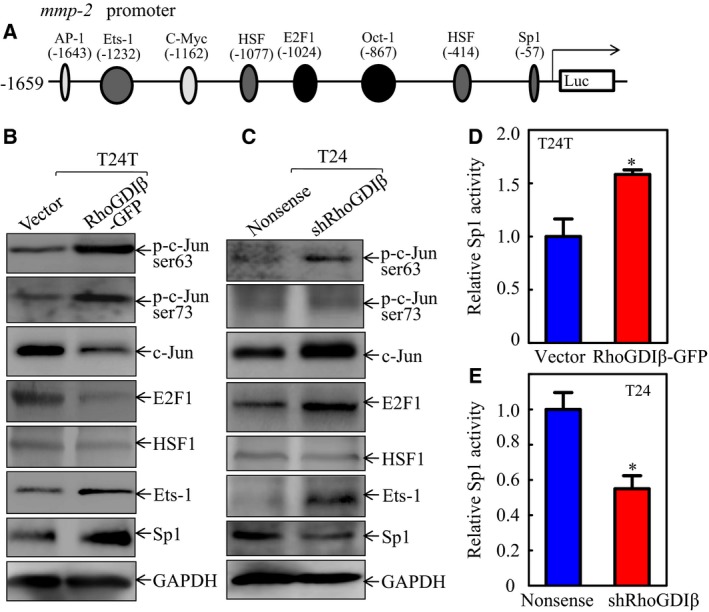
RhoGDIβ upregulated Sp1 expression and Sp1‐dependent transcription activity in human BC cells. (A) Potential transcription factor binding sites in human *mmp‐2* promoter region. (B,C) The cell extracts obtained from (B) T24T(Vector) *vs*. T24T(RhoGDIβ‐GFP) cells or (C) T24(Nonsense) *vs*. T24(shRhoGDIβ) cells were analyzed for the activation and expression of the transcription factors as indicated. (D,E) The Sp1‐dependent transcriptional activity was evaluated using Sp1‐dependent luciferase reporter. co‐transfected together with pRL‐TK into (D) T24T(Vector) and T24T(RhoGDIβ‐GFP) cells or (E) T24(Nonsense) and T24(shRhoGDIβ) cells (E). Twenty‐four hours post‐transfection, the transfectants were extracted to evaluate the luciferase activity. TK was used as an internal control. The results are presented as Sp1‐dependent activity relative to the control vector transfectant, and each bar indicates mean ± SD from three independent experiments. *Significant difference (*P *< 0.05).

Since *mmp‐2* promoter activity appeared to be upregulated by RhoGDIβ, we then tried to identify the potential transcription factors regulating *mmp‐2* promoter activity. The transcription factor binding sites of *mmp‐2* promoter are illustrated in Fig. 3A. The expressions of the transcription factors in the cells were examined. The results show that alterations of phosphorylation of c‐Jun and expression of c‐Jun, E2F1, HSF1, Ets‐1 in T24T(Vector) *vs*. T24T(RhoGDIβ‐GFP) and T24(Nonsense) *vs*. T24(shRhoGDIβ) cells were not consistent with RhoGDIβ‐regulated levels of *mmp‐2* mRNA and *mmp‐2* promoter‐driven luciferase activity (Fig. 3B, C), excluding the possibility of their involvement in RhoGDIβ‐upregulation of *mmp‐2* transcription. In contrast, levels of Sp1 were obviously increased with RhoGDIβ overexpression and decreased with RhoGDIβ downregulation (Fig. 3B, C). Moreover, Sp1‐dependent transcription activity was also increased with RhoGDIβ overexpression and decreased with RhoGDIβ downregulation (Fig. 3D, E), indicating that Sp1 may be a RhoGDIβ downstream transcription factor promoting *mmp‐2* transcription.

**Figure 4:**
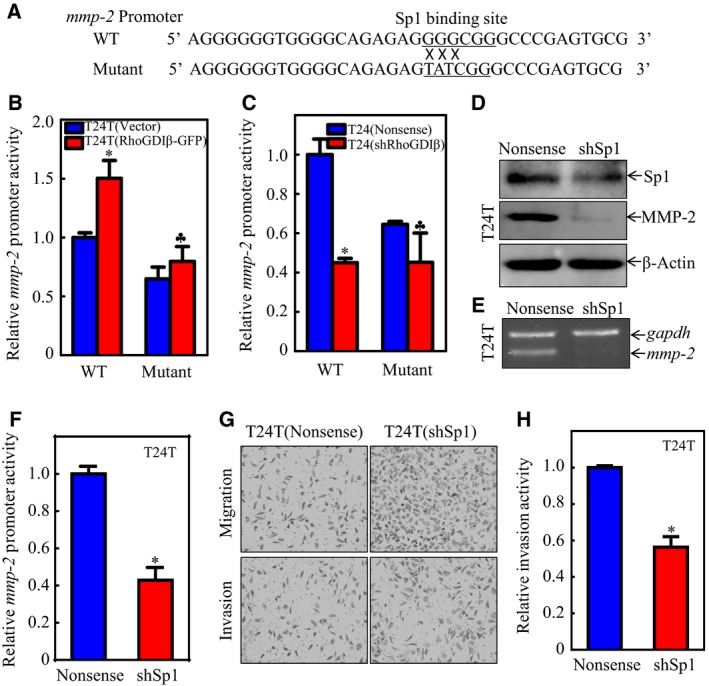
Sp1 contributed to *mmp‐2* transcription and further invasion of BC cells. (A) Illustration of the mutated Sp1 binding site in the *mmp‐2* promoter region. (B,C) Wild‐type or mutant *mmp*‐2 promoter‐driven luciferase reporters was co‐transfected together with pRL‐TK into (B) T24T(Vector) and T24T(RhoGDIβ‐GFP) cells or (C) T24(Nonsense) and T24(shRhoGDIβ) cells. Twenty‐four hours post‐transfection, the transfectants were extracted to evaluate the luciferase activity. The results were presented as *mmp‐2* promoter activity relative to the scramble vector transfectant transfected with wild‐type *mmp‐2* promoter‐driven luciferase reporter, and each bar indicates mean ± SD from three independent experiments. *Significant difference in comparison to scramble vector transfetant. ♣Significant difference in comparison with wild‐type reporter transfectant (*P *< 0.05). (D, E) The cell extracts from T24T(Nonsense) and T24(shSp1) transfectants were subjected to western blot to determine (D) MMP‐2 protein expression or (E) *mmp‐2* mRNA levels. (F) Wild‐type *mmp‐2* promoter‐driven luciferase reporter was co‐transfected together with pRL‐TK into T24T(Nonsense) and T24T(shSp1) cells, respectively. Twenty‐four hours post‐transfection, the transfectants were extracted to evaluate the luciferase activity. The results were presented as *mmp‐2* promoter activity relative to the vector control transfectants; each bar indicates mean ± SD from three independent experiments. *Significant difference (*P *< 0.05). (G,H) Invasion abilities of T24T(shSp1) and T24T(Nonsense) cells were evaluated using BD BioCoat™Matrigel™ Invasion Chamber (G). The relative invasion activity was plotted. The bars are mean ± SD from three independent experiments. *Significant difference between T24T(shSp1) and T24T(Nonsense) cells (*P* < 0.05).

To test whether Sp1 is the key RhoGDIβ downstream mediator responsible for promoting *mmp‐2* transcription, we constructed the Sp1 binding site point mutation in the *mmp‐2* promoter‐driven luciferase reporter. Both the wild‐type or mutant *mmp‐2* promoter‐driven luciferase reporters (Fig. 4A) were transfected into T24T(RhoGDIβ‐GFP) and T24(shRhoGDIβ) cells to test the *mmp‐2* promoter luciferase reporter activity. As expected, the point mutation of the Sp1 binding site impaired RhoGDIβ‐mediated *mmp‐2* promoter transcription in both cells (Fig. 4B, C). We also used shRNA, specifically targeting *sp1*, to knock down Sp1 in T24T cells; stable transfectant T24T(shSp1) was established and identified as shown in Fig. 4D. Knockdown of Sp1 in T24T cells dramatically inhibited the expressions of *mmp‐2* mRNA, MMP‐2 protein, and *mmp‐2* promoter activity, as compared with those in the scramble control transfectant, T24T(nonsense) cells (Fig. 4E, F). Consistently, the cell invasion ability was also attenuated in T24T(shSp1) cells (Fig. 4G, H). These results clearly indicate that Sp1 specifically binds to the *mmp‐2* promoter and enhances its transcription in a RhoGDIβ‐dependent fashion.

### 3.4 RhoGDIβ positively regulates JNK2 and consequently increased Sp1 protein expression and transactivation

**Figure 5:**
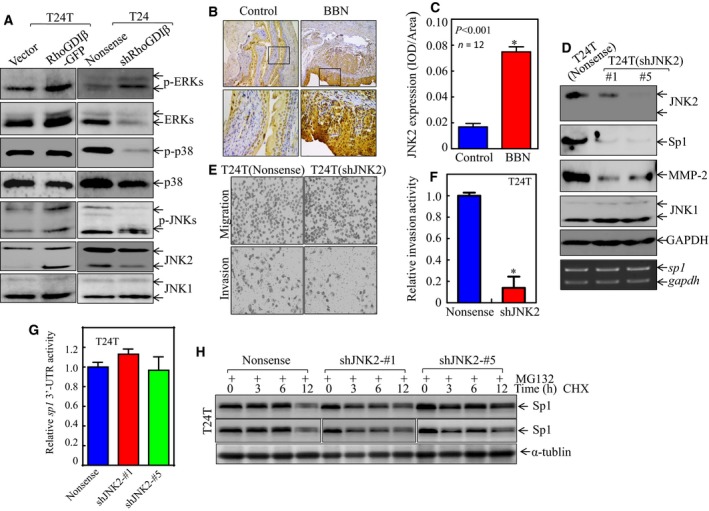
JNK2 specifically mediated Sp1 expression, in turn enhancing *mmp‐2* expression and BC cell invasion. (A) The cell extracts obtained from the transfectants, as indicated, were subjected to western blot for determination of protein expression. (B,C) IHC‐P was carried out to evaluate JNK2 protein expression in mouse BC tissues as compared with normal bladder tissues. The optical density was analyzed and calculated, as described in Materials and Methods (*n *=* *12). *Significant difference between the two groups of mice (*P *< 0.01). (D) The cell extracts obtained from the transfectants, as indicated, were subjected to western blot for determination of protein expression (top panel) or RT‐PCR for evaluation of *sp1* mRNA expression (lower panel). (E,F) Invasive abilities of T24T(shJNK2) and T24T(Nonsense) cells were determined by using the BD BioCoat™Matrigel™ Invasion Chamber. The images was captured under inverted microscopy (E) and the relative invasion activity was plotted (F). The bars are mean ± SD from three independent experiments. *Significant difference between T24T(shJNK2) and T24T(Nonsense) cells (*P *< 0.05). (G) Wild‐type *sp1* 3′‐UTR‐drived luciferase reporters were co‐transfected together with pRL‐TK into T24T(Nonsense) and T24T(shJNK2) cells, respectively. Twenty‐four hours post‐transfection, the transfectants were extracted to evaluate the luciferase activity. The results were presented as *sp1* 3′‐UTR activity relative to the vector control transfectant, and each bar indicates mean ± SD from three independent experiments. (H) Sp1 protein stability was evaluated in both MG132 pretreated T24T(Nonsense) and T24T(shJNK2) cells in the presence of 50 μg·mL ^−1^ cycloheximide (CHX). α‐Tubulin was used as a protein loading control.

To test the impact of RhoGDIβ on the regulation of Sp1 activation, we evaluated the associated ERK activation in RhoGDIβ knockdown and overexpressed T24T cells. As shown in Fig. 5A, p‐ERK was upregulated in both T24T(RhoGDIβ‐GFP) and T24(shRhoGDIβ) cells, implying that ERK is not a regulator of Sp1 (Fig. 5A). Levels of p‐p38 were dramatically decreased in T24(shRhoGDIβ) cells, but there were no observable differences between T24T(RhoGDIβ‐GFP) cells and the vector transfectant. Levels of JNK1 showed a slight increase in both T24T(RhoGDIβ‐GFP) and T24(shRhoGDIβ) cells in comparison with their respective scramble control transfectants. Interestingly, the expression of JNK2 and phosphorylated JNK2 was increased in T24T(RhoGDIβ‐GFP) cells and decreased in T24(shRhoGDIβ) cells, compared with their scramble vector transfectants (Fig. 5A), indicating that JNK2 may participate in Sp1 regulation. To test the expression level of JNK2 in bladder cancer, IHC‐P was performed to determine the JNK2 expression level in BBN‐induced invasive mouse BC, as described in Materials and Methods. The data shown in Fig. 5B, C indicate that JNK2 was dramatically elevated in mouse BC tissues, suggesting that JNK2 may play an oncogenic role in BC and may have some relationship with BC invasion.

To assess the role of JNK2 in expression of Sp1 and MMP‐2, as well as cancer cell invasion, T24T cells were transfected with the shRNA specifically targeting *jnk2*, and the knockdown transfection efficiency was analyzed (Fig. 5D). The knockdown of JNK2 expression resulted in a remarkable downregulation of Sp1 and MMP‐2 (Fig. 5D). Consistently, the knockdown of JNK2 significantly attenuated BC cell invasion (Fig. 5E, F). To investigate the molecular mechanism of how JNK2 regulates Sp1 expression, RT‐PCR was employed; the results revealed that JNK2 does not regulate *sp1* mRNA level (Fig. 5D, lower panel). The potential effect of JNK2 on *sp1* mRNA 3′‐UTR activity was further evaluated in T24T(shJNK2) cells in comparison with the scramble nonsense transfectants. The results show that there is no significant difference in 3′‐UTR activity of *sp1* in T24T(shJNK2) and T24T(Nonsense) cells (Fig. 5G), excluding the possibility of JNK2 regulation of Sp1 protein translation. Therefore, the Sp1 protein degradation rate was evaluated in T24T(shJNK2) cells and T24T(nonsense) cells in the presence of cycloheximide (CHX) to block new protein synthesis. The results indicated that Sp1 degradation in T24T(shJNK2) cells was much faster than in T24T(nonsense) cells (Fig. 5H). These results demonstrate that RhoGDIβ upregulated Sp1 protein stability, consequently promoting MMP‐2‐mediated BC cell invasion.

**Figure 6:**
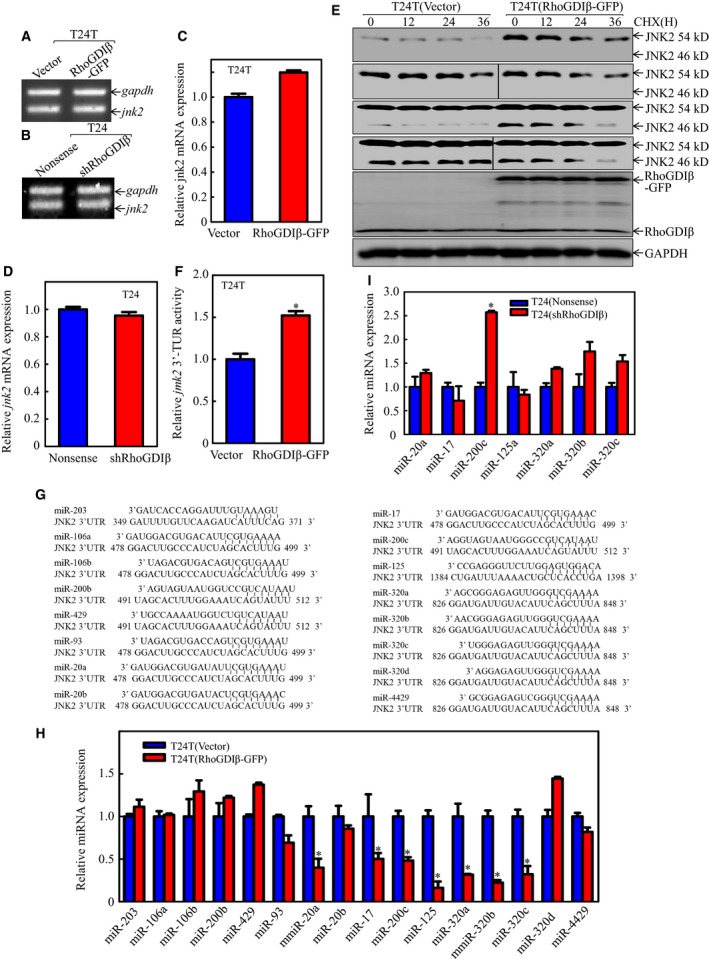
RhoGDIβ specifically inhibited miR‐200c expression and promoted JNK2 protein expression in human BC cells. (A‐D) The *jnk2* mRNA levels were analyzed by RT‐PCR or real‐time PCR in (A,C) T24T(Vector) *vs*. T24T(RhoGDIβ‐GFP) cells or (B,D) T24(Nonsense) *vs*. T24(shRhoGDIβ) cells. (E) After treatment of T24T(Vector) and T24T(RhoGDIβ‐GFP) cells with 50 μg·mL ^−1^ cycloheximide (CHX), JNK2 protein stability was evaluated at time points as indicated. GAPDH was used as a protein loading control. (F) Wild‐type *jnk2* 3′‐UTR luciferase reporter was co‐transfected together with pRL‐TK into T24T(Vector) and T24T(RhoGDIβ‐GFP) cells. Twenty‐four hours post‐transfection, the transfectants were extracted to evaluate the luciferase activity. TK was used as an internal control. The results are presented as *jnk2* 3′‐UTR activity relative to the control vector transfectant, and each bar indicates mean ± SD from three independent experiments. *Significant difference (*P *< 0.05). (G) The potential miRNA binding sites in the 3′‐UTR region of *jnk2* mRNA. (H,I) The expression levels of miRNA were evaluated by real‐time PCR in (H) T24T(Vector) *vs*. T24T(RhoGDIβ‐GFP) cells or (I) T24(Nonsense) *vs*. T24(shRhoGDIβ) cells. The results were normalized to U6.

To explore how RhoGDIβ promotes JNK2 abundance at the protein level, RT‐PCR was performed to compare the expression levels of *jnk2* mRNA in T24T(RhoGDIβ‐GFP) and T24(shRhoGDIβ) cells. Interestingly, the level of *jnk2* mRNA did not show an observable change between two transfectants (Fig. 6A, D). This led us to examine whether RhoGDIβ regulated JNK2 protein degradation. CHX (a protein synthesis inhibitor) was added to the cell cultures to prevent new protein synthesis, and JNK2 protein degradation in the T24T(RhoGDIβ‐GFP) transfectants and T24T(Vector) transfectants was evaluated (Fig. 6E). The level of JNK2 at 54 kD in T24T(RhoGDIβ‐GFP) cells was comparable with that observed in the scramble control transfectants. However, the 46 kD of JNK2 in T24T(RhoGDIβ‐GFP) cells had a shorter half‐life (Fig. 6E), which is not consistent with the increased JNK2 protein level in T24T(RhoGDIβ‐GFP) cells. Thus, we anticipate that RhoGDIβ promotes JNK2 protein translation.

### 3.5 RhoGDIβ inhibited transcription of miR200c, releasing miR200c binding to the 3′‐UTR of *jnk2* mRNA and increasing JNK2 protein translation

**Figure 7:**
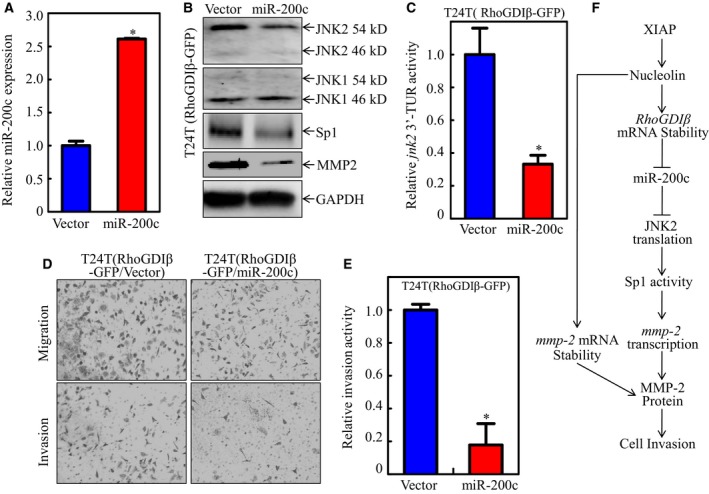
miR200c targeted *jnk2* 3′‐UTR, consequently resulting in blockage of JNK2 protein translation and inhibition of invasion of BC cells. (A) Real‐time PCR was used to identify the miR‐200c expression in T24T(RhoGDIβ/miR‐200c) cells in comparison with T24T (RhoGDIβ/Vector) cells. (B) The cell extracts from T24T(RhoGDIβ/miR‐200c) and T24T(RhoGDIβ/Vector) cells were used to evaluate the effect of miR‐200c on expression of JNK1, JNK2, Sp1, and MMP‐2. GAPDH was used as a protein loading control. (C) The *jnk*2 3′‐UTR activity was evaluated by transfection of *jnk2* 3′‐UTR‐driven luciferase reporter together with pRL‐TK into the transfectants as indicated. The bars indicate mean ± SD from three independent experiments. *Significant difference (*P *< 0.05). (D,E) Invasive abilities of T24T(RhoGDIβ‐GFP/miR‐200c) and T24T/(RhoGDIβ/Vector) were analyzed using a BD BioCoat™Matrigel™ Invasion Chamber. The images were captured under inverted microscopy (D) and the invasion activity was analyzed and plotted (E). The bars are mean ± SD from three independent experiments. *Significant difference between the two transfectants (*P *< 0.05). (F) XIAP/RhoGDI overexpression leading to MMP‐2 overexpression and invasion in human BC cells.

To test whether RhoGDIβ affects JNK2 protein translation, we introduced *jnk2* 3′‐UTR‐driven luciferase reporter into T24T(RhoGDIβ**‐**GFP) cells. The results showed that ectopic expression of RhoGDIβ significantly increased in *jnk2* 3′‐UTR luciferase activity (Fig. 6F). We next used the ‘targetscan.org’ website to analyze the potential miRNA binding sites in *jnk2* 3′‐UTR luciferase reporter (Fig. 6G). Real‐time PCR was performed to evaluate the relative expression levels of these miRNAs in T24T(Vector) *vs*. T24T(RhoGDIβ‐GFP) cells. The results indicated that seven miRNAs were downregulated in T24T(RhoGDIβ‐GFP) cells as compared with the vector transfectants (Fig. 6H). We further evaluated the expression levels of those miRNAs in T24(Nonsense) *vs*. T24(shRhoGDIβ) cells and found that only miR‐200c abundance was increased in T24(shRhoGDIβ) cells (Fig. 6I), suggesting that miR200c may be involved in RhoGDIβ modulation of JNK2 expression. To test this notion, miR‐200c was overexpressed in T24T(RhoGDIβ‐GFP) cells and the stable transfectants were identified (Fig. 7A). Following the ectopic expression of miR‐200c, JNK2, but not JNK1, was inhibited (Fig. 7B). Concomitantly, Sp1 and MMP‐2 expression was also impaired (Fig. 7B). In addition, the *jnk2* mRNA 3′‐UTR activity was attenuated in miR‐200c overexpressed cells (Fig. 7C). Moreover, the results from an invasion assay indicated that overexpression of miR‐200c dramatically suppressed the invasive ability of T24T(RhoGDIβ) cells as compared with that in the scramble vector transfectant (Fig. 7D, E). These results imply that RhoGDIβ overexpression inhibits miR‐200c abundance, which results in promoting JNK2 protein translation and subsequently upregulating Sp1 activity and *mmp‐2* transcription, as well as promoting BC cell invasion.

## 4 Discussion

Muscle invasive bladder cancers can progress to life‐threatening metastases; the 5‐year overall survival rate in patients with distant metastasis is 6% (Choi *et al*., 2014a). Discovering and understanding the underlying mechanisms of BC invasion would be a key step towards finding novel therapeutic approaches to treat this malignant disease. The function of RhoGDIβ in regulating cancer cell motility varies in different types of cancer cells, depending on the types and the stage of cancer, as well as the downstream mediators or effectors (Cho *et al*., 2014b; Fang *et al*., 2014; Niu *et al*., 2015; Yi *et al*., 2015). It has been reported that RhoGDIβ possesses an inhibitory function for cancer invasion and metastasis in human bladder and lung cancers (Gildea *et al*., 2002); however, it is also been found to promote gastric cancer metastasis through upregulation of VEGF‐C (Cho *et al*., 2014a). Our most recent studies show that RhoGDIβ is overexpressed in most human BC and in 100% of BBN‐induced invasive mouse BC, and we further demonstrate that RhoGDIβ overexpression is associated with promotion of human BC invasion *in vitro* and lung metastasis *in vivo* (Y. Yu and C. Huang, unpublished data). In the present study, using the strategies of gain (overexpression)‐ or loss (knockdown)‐function approaches, we show that overexpressed RhoGDIβ inhibits miR‐200c expression, releasing its binding to the 3′‐UTR region of *jnk2* mRNA and promoting JNK2 expression, in turn leading to Sp1 activation and *mmp‐2* transcription, as well as promoting human bladder cancer cell invasion. Although we are still exploring the molecular basis for the differential functions of RhoGDIβ in BC, we anticipate that the differences are due to various stages of BC cells. This notion is strongly supported by our most recent data showing that RhoGDIβ shows an inhibitory effect on BBN‐treated urothelial cell growth during early treatment, but plays a promotive effect on BBN‐treated normal human urothelial cells when the cells have been treated over 15 weeks (X. Hua *et al*., data not shown).

Cancer metastasis involves multiple steps, including local tumor cell invasion, entry into the vasculature, exit of carcinoma cells from circulation, and colonization at the distal sites (van Zijl *et al*., 2011). Stromal invasion is a key step in cancer cell invasion that involves the destruction of the basement membrane to the linked tissue (Watanabe, 2010). MMP‐2 and MMP‐9 are two major MMPs that play a central role in extracellular matrix digestion by hydrolyzing type IV collagen, which is the main component of the extracellular matrix. It is well known that MMP‐2 and MMP‐9 are overexpressed and promote cancer cell invasion and metastasis in many cancers (Chen *et al*., 2012; Kenny *et al*., 2008; Lee *et al*., 2008; Li *et al*., 2013; Roomi *et al*., 2009; Samantaray *et al*., 2004; Yan *et al*., 2011). For example, MMP‐2 overexpression is required for breast cancer and brain metastasis (Mendes *et al*., 2007). The levels of MMP‐2 and MMP‐9 expression are considered biomarkers for judging the malignancy of prostate cancer progression and evaluating therapeutic effects (Morgia *et al*., 2005). MMP‐2 expression has been reported to be regulated by ERK (Kuo *et al*., 2006; Mendes *et al*., 2007), through which *mmp‐2* mRNA stability was increased upon irradiation (Zhao *et al*., 2004). Our published studies show that MMP‐2, but not MMP‐9, is specifically responsible for BC invasion through nucleolin stabilization of *mmp‐2* mRNA. In the present study, we found that the MMP‐2 expression level correlates well with RhoGDIβ expression and cell invasion ability. We also show that the knockdown of MMP‐2 impaired the invasive ability of T24T(RhoGDIβ‐GFP) cells. These findings suggest that MMP‐2 is crucial for RhoGDIβ to exert its positive effect on BC invasion. Our experiments further indicate that Sp1 expression and activity are increased in RhoGDIβ overexpressed cells. Through binding to the *mmp‐2* promoter, Sp1 upregulated *mmp‐2* transcription and expression, further promoting the invasive ability of BC cells.

Transcription factor Sp1 is a zinc finger transcription factor that binds to GC‐rich motifs of promoters and is involved in the regulation of diverse cellular processes such as cell differentiation, cell growth, apoptosis, DNA damage, and chromatin remodeling. It has been shown that Sp1 acts as a tumor suppressor to suppress colorectal cancer growth and metastasis by increasing miRNA‐52d‐5p transcription for target CTHRC1 (Yan *et al*., 2015). We have reported that the anti‐cancer drug ISO downregulates Sp1 translation to block BC cell growth and IHC‐P results show Sp1 is upregulated in BBN‐induced BC tissues, suggesting an oncogenic role of Sp1 in BC (Zeng *et al*., 2016). Here, we further reveal that Sp1 bound directly to the *mmp‐2* promoter and increased its transcription, promoting bladder cancer cell invasion. Sp1 is regulated through various modifications (such as phosphorylation, acetylation, glycosylation, and proteolysis) (Wormke *et al*., 2003). MAP kinase (MAPK) has been reported to regulate Sp1 activity (Benasciutti *et al*., 2004; Chuang *et al*., 2008). In our current studies, we analyzed the levels of various intracellular signaling regulators, such as JNK1, JNK2, ERK, and p38, in T24T(Vector) *vs*. T24T(RhoGDIβ), and T24(Nonsense) *vs*. T24(shRhoGDIβ) cells. The results indicate that JNK2 is responsible for the upregulation of Sp1 by RhoGDIβ. By measuring *sp1* mRNA levels, *sp1* 3′‐UTR activity, and Sp1 protein stability, we also demonstrate that Sp1 is regulated at the protein degradation level.

MiR‐200c is a member of the miR‐200 family, which contains miR‐200a, miR‐200b, miR‐141, and miR‐429 (Korpal *et al*., 2008). These five miRNAs are all involved in regulating cancer metastasis (Dykxhoorn, 2010; Gregory *et al*., 2008). miR‐200c also acts as a considerable modulator in the process of epithelial‐to‐mesenchymal transition (EMT), which affects tumor development and metastasis (Hur *et al*., 2013; Liu *et al*., 2014). It has been reported that miR‐200c inhibits MMP‐3 expression, which in turn abolishes ovarian cancer metastasis (Sun *et al*., 2014), downregulates E‐cadherin, and affects the EMT in human renal cell carcinoma (Wang *et al*., 2013), as well as targeting the 3′‐UTR region of *jnk2* mRNA to inhibit the metastasis of colorectal cancer (Sui *et al*., 2014). It has been reported that miR‐200c has an oncogenic function in bladder cancer (Cheng *et al*., 2016; Mahdavinezhad *et al*., 2015a,b). MiR‐200c is also reported to promote BC cell invasion by directly targeting the gene of reversion inducing cysteine‐rich protein with kazal motifs (RECK) by application of oligonucleotides of anti‐miR‐200c to EJ and T24 cells (Cheng *et al*., 2016). Our present study showed that miR‐200c expression was downregulated in RhoGDIβ overexpressed cells and enhanced in RhoGDIβ knockdown cells. We also found that miR‐200c was able to inhibit BC cell invasion in metastatic T24T cells. MiR‐200c was further demonstrated directly to target the 3′‐UTR of *jnk2*, which consequently resulted in interference with JNK2 protein translation. JNK2 has been reported to mediate invasion of many types of cancers, including glioblastoma cancer (Saunders *et al*., 2015), breast cancer (Saunders *et al*., 2015), and colorectal cancer (Sui *et al*., 2014). Our present study reveals a new function of miR‐200c in the regulation of JNK2 protein translation in metastatic BC cells and also provides a new role for JNK2 in the regulation of BC invasion.

In summary, we find that RhoGDIβ promotes BC invasion by upregulating MMP‐2 expression. We demonstrate that Sp1 is a critical factor in regulating *mmp‐2* transcription in our experimental setting, and RhoGDIβ, via inhibition of miR‐200c, increase in JNK2 protein translation, and Sp1 activity for promotion of BC invasion (Fig. 7F). Our studies provide highly significant insights into understanding the nature of RhoGDIβ in BC invasion and its development.

## Author contributions

CH and HH were involved in the design of the study. HH, HJ, HZ, JW, XL, HY, SW, XG, LX, JL, MP, AW, and JZ carried out the experiments and acquired data (cell culturing and mouse breeding, gene expression analysis, etc). CH, HH, and HJ analyzed the data and drafted the manuscript. JL, X‐RW, and CC provided administrative, technical, or material support. CH supervised the study and all authors reviewed and revised the manuscript.
